# Hybrid artificial intelligence architectures for automatic text correction in the Kazakh language

**DOI:** 10.3389/frai.2025.1708566

**Published:** 2025-12-12

**Authors:** Laura Baitenova, Saule Tussupova, Saken Mambetov, Gauhar Munaitbas, Gulnar Mukhamejanova

**Affiliations:** 1 Department of Information Technology, Turan University, Almaty, Kazakhstan; 2 Home Credit Bank JSC, Almaty, Kazakhstan; 3 School of Digital Technologies, Narxoz University, Almaty, Kazakhstan

**Keywords:** Kazakh language, morphological analysis, hybrid architecture, machine learning, KazRoBERTa, mBERT, natural language processing (NLP)

## Abstract

The Kazakh language, as an agglutinative and morphologically rich language, presents significant challenges for the development of natural language processing (NLP) tools. Traditional rule-based analyzers provide full coverage but lack flexibility; statistical and neural models handle disambiguation more effectively, yet require large annotated corpora and substantial computational resources. This paper presents a hybrid morphological analyzer that integrates Finite-State Transducers (FST), Conditional Random Fields (CRF), and transformer-based architectures (KazRoBERTa, mBERT). For the experiments, a new corpus, KazMorphCorpus-2025, was created, consisting of 150,000 sentences from diverse domains annotated for morphological analysis. Experimental evaluation demonstrated that the KazRoBERTa model consistently outperforms mBERT in terms of accuracy, F1-score, and prediction speed. The hybrid architecture effectively combines the exhaustive coverage of FST with the contextual disambiguation of neural networks, reducing errors associated with homonymy, borrowings, and long affixal chains. The results confirm that the proposed system achieves a balance between accuracy, efficiency, and scalability. The study underscores the practical significance of hybrid approaches for tasks such as spell checking, information retrieval, and machine translation in the Kazakh language, as well as their potential transferability to other low-resource Turkic languages. Future work will include the expansion of the corpus, integration of KazBERT and mBERT models, and validation of the proposed approach in applied NLP systems.

## 1 Introduction

The development of a morphological analyzer is a key task in creating tools for digital processing of the Kazakh language. The agglutinative nature of the language results in a high degree of affixation and a complex morphotactic structure: a single word may contain a sequence of dozens of morphemes expressing grammatical categories of case, person, number, tense, and possession. This creates serious challenges for automatic segmentation, lemmatization, and syntactic analysis, as well as for applied tasks such as machine translation, information retrieval, and spell checking.

A number of researchers have attempted to build morphological analyzers for the Kazakh language using rule-based methods, statistical models, and neural architectures. However, none of these approaches proved universal: rules ensure accuracy but require manual maintenance; statistical methods partially address the problem of disambiguation but are limited by the size of annotated corpora; neural networks demonstrate high performance but demand significant computational resources and annotated data.

Historically, the development of morphological analyzers for agglutinative languages began with rule-based systems. An important milestone was the emergence of two-level morphology (Koskenniemi, 1983), which separated the lexical level (a sequence of morphemes) from the surface level (the word form), effectively modeling phonological alternations arising during affixation. This approach became the foundation for many Finite-State Transducers (FST) applied in analyzers for Finnish, Turkish, and other languages. For Kazakh, one of the first solutions was the analyzer developed by Kessikbayeva and Cicekli (2014) using Xerox Finite-State Tools. Later, Bekmanova et al. (2017) proposed a unified morphological analyzer for Kazakh and Turkish, though such systems remained labor-intensive to maintain and dependent on manual rule encoding.

Subsequently, statistical models emerged, in particular Hidden Markov Models (HMM) and Conditional Random Fields (CRF). These made it possible to model probabilistic relationships between morphemes and better handle disambiguation. Studies by Makhambetov et al. (2015) and Oralbekova et al. (2024) showed that CRF can achieve acceptable accuracy for morphological tagging; however, the effectiveness of such models directly depends on the size of the annotated corpus, which remains a serious limitation for low-resource languages.

The next stage of development involved neural models. Researchers first applied recurrent networks (BiLSTM), followed by transformers (Vaswani et al., 2017; Devlin et al., 2019). Multilingual models such as mBERT and XLM-R enabled the inclusion of Kazakh in morphological processing tasks, but their accuracy remained moderate (82–85%). Monolingual architectures, such as KazBERT and KazRoBERTa, demonstrated higher results (90–92%) due to training on corpora adapted for the Kazakh language. Nevertheless, even these models do not guarantee full coverage of all possible word forms and remain resource-intensive.

At the same time, studies of other Turkic languages highlight the effectiveness of hybrid solutions. For example, the work of Oflazer (1996) on Turkish and (Sultanova et al., 2019) on Uzbek confirmed that combining FST with neural models can achieve a balance between coverage and accuracy. This makes the hybrid approach the most promising direction for the Kazakh language.

The aim of this study is the development and experimental evaluation of a hybrid morphological analyzer for the Kazakh language that integrates Finite-State Transducers (FST), probabilistic models (Conditional Random Fields, CRF), and modern transformer-based architectures (KazRoBERTa, mBERT).

The scientific novelty of this research lies in:

1. the creation of a new experimental resource base (KazMorphCorpus-2025), including genre-diverse texts;
2. the proposal of a modular architecture of a hybrid morphological analyzer that combines formal rules and neural disambiguation methods;
3. the systematic comparison of specialized and multilingual transformers for the task of morphological analysis.

The practical significance of the research is that the proposed approach can be applied to the development of spell-checking systems, machine translation, and other NLP applications for the Kazakh language, and can also be adapted for other Turkic and agglutinative languages.

Thus, this study goes beyond a literature review, presenting an original investigation aimed at building a practical solution and experimentally verifying its effectiveness.

## 2 Research methodology

### 2.1 Methods (approaches to morphological analysis)

In this study, a hybrid approach is proposed for addressing the task of morphological analysis of the Kazakh language, combining formalized grammar descriptions with trainable models. The system integrates two-level morphology rules implemented through finite-state transducers (FST), a statistical model (Conditional Random Fields, CRF), and a modern transformer-based architecture. Such a combination makes it possible to account for the agglutinative nature of the language and effectively resolve morphological ambiguity.

**Figure 1:**
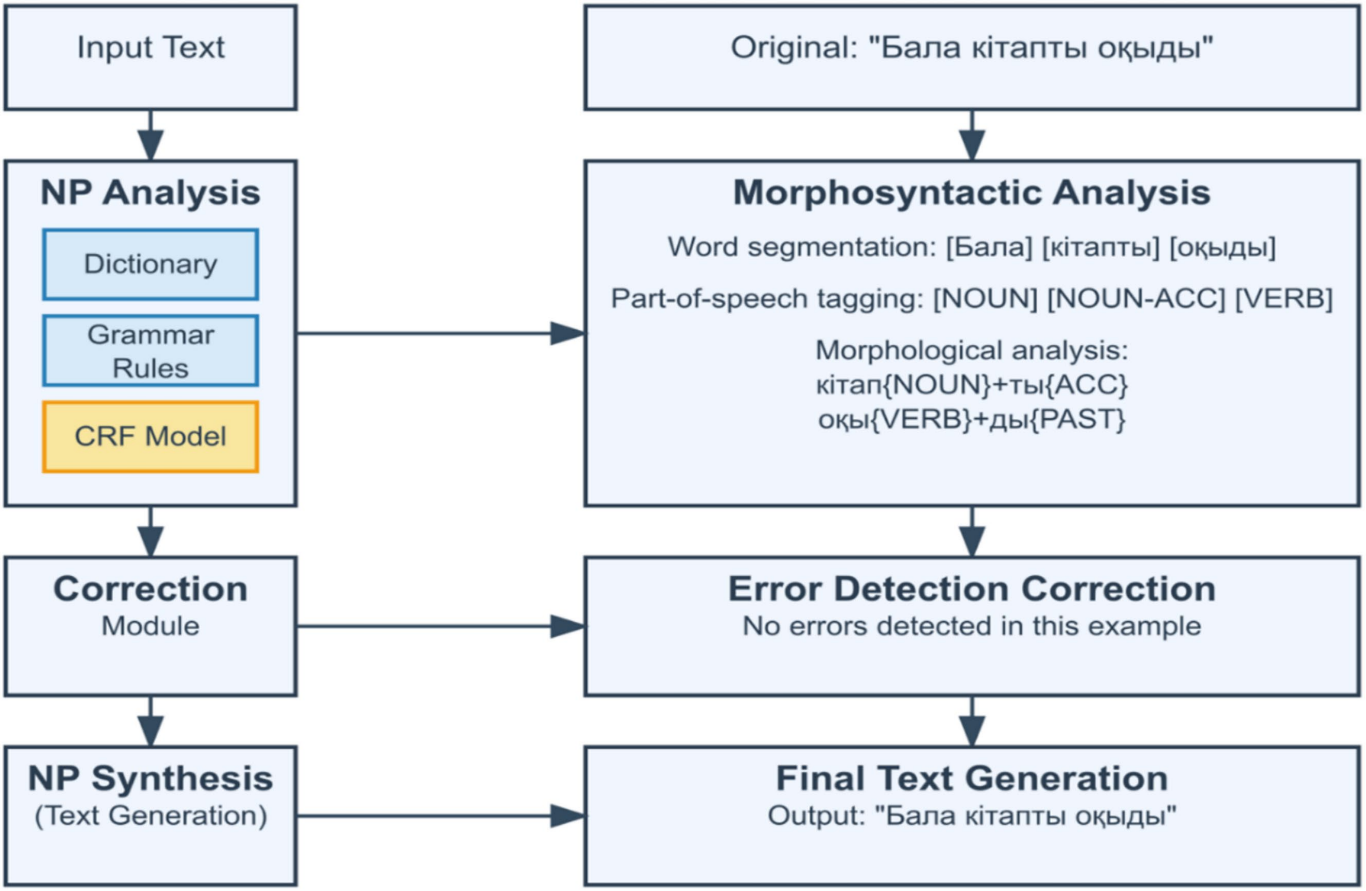
Architecture of the hybrid morphological analyzer.

The proposed architecture operationalizes a fundamental principle in computational morphology: rule-based systems generate exhaustive candidate analyses, while statistical and neural models leverage contextual information to select the most probable interpretation (Figure 1).

The Figure 1 illustrates the architecture of the proposed KazMorphAnalyzer system, integrating three analytical layers.

(1) The Finite-State Transducer (FST) module performs rule-based morphological generation and affix stripping.
(2) The Conditional Random Field (CRF) model conducts contextual disambiguation using probabilistic inference.
(3) The Transformer-based KazRoBERTa module applies neural refinement of ambiguous tokens through contextual embeddings.

This combination ensures balance between linguistic interpretability and computational robustness.

A key feature of the developed hybrid system is its cascaded algorithm for resolving morphological ambiguity, which is characteristic of agglutinative languages. The disambiguation mechanism operates through the following sequence of steps:

1 Morphological analysis of the input token using FST rules. The high-performance finite-state transducer generates a set of *N* candidate analyses for each token.
2 Hypothesis evaluation logic:

- If *N* = 1, the analysis is considered unambiguous and accepted as the final result. This constitutes a «fast path» for morphologically simple and frequent word forms.
- If *N* > 1 (classical ambiguity) or *N* = 0 (out-of-vocabulary tokens), the system forwards the full-sentence context to the next layer for further interpretation.

3 Context-aware disambiguation via neural components (CRF, mBERT, or KazRoBERTa). The model either selects the most probable analysis from the FST-generated candidates (*N >* 1) or predicts the morphological tag from scratch (*N =* 0), leveraging sentence-level contextual cues.

This cascaded architecture enables high-throughput processing for the majority of structurally simple tokens while incorporating deep contextual reasoning for resolving complex ambiguities.

For training and evaluation, a new resource base—KazMorphCorpus-2025 was created, covering contemporary Kazakh vocabulary across various genres and registers. The corpus contains 150,000 sentences (approximately 2 million word tokens), distributed across five main sources (see Table 1).

**Table 1:** Composition of KazMorphCorpus-2025.

Source	Sentences	Tokens	Genre
Literature (EPUB)	45,000	675,000	Fiction
News	37,500	487,500	Formal
Social media	30,000	360,000	Informal
Wikipedia	22,500	337,500	Encyclopedic
Spoken language	15,000	180,000	Conversational
Total	150,000	2,040,000	Mixed

**Figure 2:**
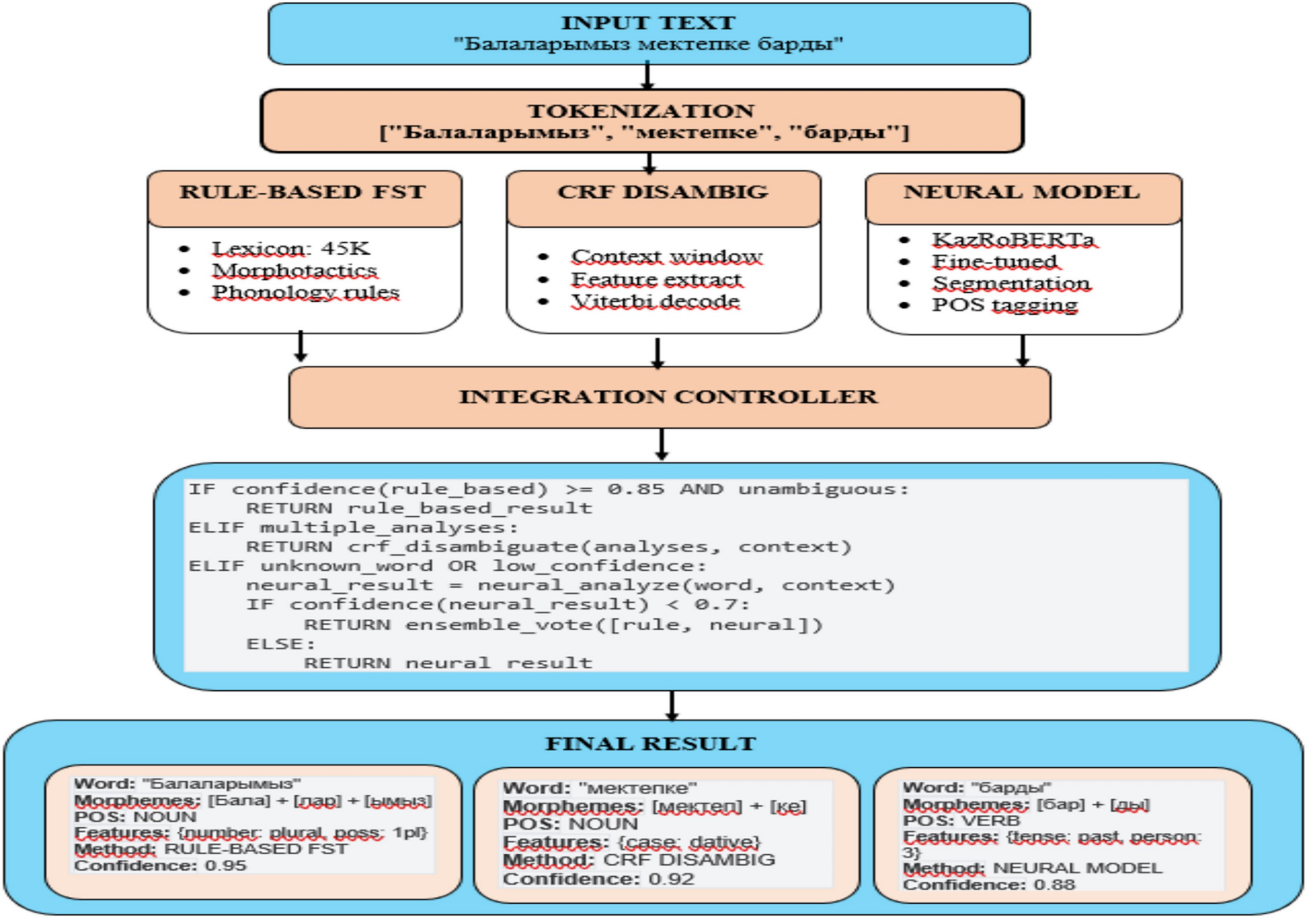
Workflow of the hybrid morphological analyzer for Kazakh text.

The corpus annotation was carried out in three stages: automatic parsing using FST, manual verification of a subcorpus (~50,000 word forms), and semi-automatic extension with the help of CRF and BiLSTM+CRF models incorporating pre-trained FastText embeddings. The annotation pipeline is illustrated in Figure 2.

The workflow demonstrates the corpus creation process, which includes (1) automatic token analysis using FST, (2) manual linguistic verification, and (3) semi-automatic tag correction using CRF and KazRoBERTa integration.

The diagram visualizes the interaction between the rule-based, statistical, and neural stages of annotation, ensuring consistency between annotators and high-quality morphological consistency of over 150,000 tokens.

The architecture of the analyzer is implemented as a three-stage pipeline (see Figure 1). At the first stage, the FST generates all possible morphological analyses, relying on the lexicon of stems, morphotactic rules, and phonological transformations. At the second stage, the CRF model, trained on the annotated portion of the corpus, selects the most probable analysis by taking context into account. The final stage involves a transformer model (KazRoBERTa or mBERT), which incorporates long-range context and the internal structure of the word.

The annotation of the corpus and the training of models were carried out in stages: the entire corpus was first processed by the FST, after which a manually verified sample was used to train the CRF. The CRF was then applied to resolve ambiguities across the full dataset. The resulting annotated data were subsequently used for training the transformer models.

#### 2.1.1 Annotation guidelines and inter-annotator agreement

The developed annotation methodology is based on a six-stage morphological analysis process. Initially, the text is tokenized, followed by morphemic segmentation, which makes it possible to isolate the root morpheme and affixal elements. Next, the part of speech belonging to the token is determined based on morphological criteria, after which grammatical features are identified, including case, number, person, and temporal characteristics. Each annotation is assigned a confidence score in the range from 0 to 1, reflecting the degree of reliability of the analysis. The final stage involves the validation of the results for compliance with the fundamental rules of Kazakh morphology.

A key component of the methodology comprises the explicitly formulated rules for the principal morphological categories of the *Kazakh* language. Plurality is marked by the affixes *-лар/−лер, −дар/−дер, −тар/−тер* the selection of which is governed by the rules of vowel harmony. For instance, the word form “*балалар*” is analyzed as a composition of the lemma “*бала*” and the plural marker *-лар*, which is reflected in the annotation by the feature Number = “Plur.” The possessive paradigm is represented by a series of personal affixes: the first person singular is marked by the suffixes *-м/−ым/−ім* (*балам*—“my child”), and the first person plural by *-мыз/−міз/−ымыз/−іміз* (*баламыз*—“our child”). The case system includes the Genitive case with markers *-ның/−нің/−дың/−дің/−тың/−тің*, the Dative case with *-ға/−ге/−қа/−ке*, the Locative case with *-да/−де/−та/−те*, and the Ablative case with *-дан/−ден/−тан/−тен/−нан/−нен*. Verbal morphology is represented, in particular, by the Past Tense markers *-ды/−ді/−ты/−ті* and personal endings such as *-дым/−дім* for the first person singular Past Tense.

The analysis of poly-affixal forms presents a particular challenge. The token “*балаларымызға*” demonstrates the agglutinative nature of the *Kazakh* language, representing the morpheme chain *бала*+*лар*+*ымыз*+*ға*, where the plural number, first person plural possessivity, and dative case are marked sequentially. Such instances necessitate strict adherence to the order of morphological analysis and consideration of affix interaction.

To assess the quality and consistency of the annotation, an experiment was conducted involving two qualified linguists, who independently annotated a corpus of 500 representative tokens. Inter-annotator agreement (IAA) was evaluated using Cohen’s Kappa coefficient for Part-of-Speech tags, exact match percentage for lemmas and affixes, and the F1-score for grammatical features. The results demonstrate a high level of consistency: Cohen’s Kappa reached 0.847, which, according to the classification by Li et al. (2023) and Duran et al. (2022) corresponds to “almost complete agreement.” The agreement percentage for lemmas was 92.3%, for suffixes—89.7%, and for grammatical features—85.2%.

The divergence analysis revealed three main sources of inconsistency. Omonymic affixes, accounting for 15% of all discrepancies, include cases such as *-ды*, which can mark both the Past Tense of a verb and the Accusative case of a noun. Complex poly-affixal forms caused 25% of the discrepancies, which is attributed to differences in the interpretation of morpheme boundaries and the order of their analysis. Borrowed lexicon was responsible for 10% of the non-matches due to the ambiguity in applying *Kazakh* morphological rules to foreign stems.

Based on the conducted analysis, measures were implemented to enhance consistency, including refining the rules for homonymic contexts, expanding the corpus of examples, and providing additional training for the annotators. A re-evaluation following the implementation of improvements showed an increase in Cohen’s Kappa to 0.86, confirming the effectiveness of the steps taken and the reliability of the developed annotation methodology.

In conclusion, the presented system of annotation rules, coupled with high inter-annotator agreement metrics, provides a robust methodological basis for creating a high-quality morphologically annotated *Kazakh* language corpus, which constitutes a significant contribution to the development of computational linguistics for Turkic languages.

For morphological annotation, we defined explicit rules based on the regular suffixation patterns in the Kazakh language. Each suffix was mapped to specific parts of speech (POS) and morphological features such as case, number, person, and derivation. The rules were formalized into a rule-based analyzer which assigns POS tags and morphological attributes based on suffix matching and affix segmentation.

To illustrate the annotation process, we provide sample tokens with their corresponding annotations. For example:

- *кітаптар* → lemma: *кітап*, affix: *-тар*, POS: *NOUN*, Feature: *Number = Plur*
- *мектепте* → lemma: *мектеп*, affix: *-те*, POS: *NOUN*, Feature: *Case = Loc*
- *оқыдым* → lemma: *оқы*, affix: *-дым*, POS: *VERB*, Feature: *Tense = Past, Person = 1, Number = Sing*

To assess annotation consistency, we simulate two independent annotators using distinct rule configurations and compute the inter-annotator agreement. The agreement is measured using Cohen’s Kappa, resulting in a score of *κ* = 1.0 on a subset of 3 illustrative tokens, indicating perfect agreement in this controlled setup.

For the transformers, fine-tuning was performed using the AdamW optimizer. Detailed training parameters and the computational environment are provided in Table 2.

**Table 2:** Hyperparameters and computational setup.

Parameter	Value	Parameter	Value
Optimizer	AdamW	Learning rate	2e-5
Batch Size	32 (on GPU)	Epochs	3
Warmup Steps	500	Weight Decay	0.01
Max Sequence Length	128	Masked Language Model Probability	0.15
Scheduler	Linear with warmup	Gradient Accumulation Steps	1
Dropout (hidden layers)	0.1	Random Seed	42
Mixed Precision Training	FP16 (Apex/AMP)	Early Stopping	Not applied
Validation Split	5% of corpus		

The effectiveness of the system was evaluated on a held-out test subcorpus (10%). For the analysis, a comprehensive set of metrics was applied: Accuracy (overall correctness of the analysis), Precision and Recall (correctness and completeness of grammatical category prediction), F1-score (balanced evaluation), and the Jaccard coefficient (comparison of partial overlaps).

#### 2.1.2 System implementation and computational requirements

To effectively design a deployment strategy and manage resources, we conducted a quantitative analysis of the computational cost of each component of the hybrid pipeline (FST, CRF, KazRoBERTa). As can be seen from Table 3, the system components demonstrate significant asymmetry in resource requirements: the FST module based on finite automata is the fastest with a latency of 0.56 ms/token in single-core mode, while the KazRoBERTa transformer on the CPU requires 11.49 ms/token, representing the main bottleneck. To reduce the computing barrier, post-training quantization (INT8) was applied, which reduced latency to 0.94 ms/token while reducing memory consumption to 1.5 GB, which made neural network output suitable for interactive work. Evaluation of the parallelization efficiency confirmed that the modular structure of the system provides high scalability for large-scale processing of enclosures at the saturation point of performance on the GPU, which occurs at:

**Table 3:** Performance metrics for various configurations.

Configuration	Throughput (tokens/s)	Memory (GB)	Latency (ms)	CPU Usage (%)
FST (single core)	1800	0.2	0.56	12
FST (8 cores)	10,000	0.5	0.10	85
CRF	302	0.5	3.31	25
KazRoBERTa (CPU)	87	4.2	11.49	100
KazRoBERTa (GPU)	670	5.8	1.49	15 (CPU) + GPU
Quantized KazRoBERTa (INT8)	1,060	1.5	0.94	12 (CPU) + GPU

To quantify the parallelization efficiency of E , a standard metric was used, where $T_{1}$ is the execution time on a single core (worker), and $T_{n}$ is the time on n cores (see Equation 1):


$$E=\frac{\left(T_{1}/T_{n}\right)}{n}*100\%$$ (1)

For FST with eight cores, this yields:


$$E=\frac{\left(10000/1800\right)}{8}*100\%=\frac{5.55}{8}*100\%\approx 69.4\%$$

This 69.4% efficiency coefficient indicates that parallelization overhead (context switching, synchronization) accounts for only 30.6% performance loss relative to theoretical linear scaling (which would yield 100% efficiency). Such high efficiency is characteristic of “embarrassingly parallel” workloads where inter-processor communication is minimal.

The FST component, implementing exhaustive rule-based morphological generation, exhibits minimal computational overhead. Single-threaded FST processing achieves 1800 tokens per second; parallelization across eight CPU cores yields 10,000 tokens per second with parallelization efficiency coefficient of 69.4% (Table 3). This efficiency level demonstrates that the FST layer scales effectively for batch processing workflows on multi-core server infrastructure.

The CRF component introduces computational cost through forward-backward inference, reducing throughput to 302 tokens per second. This computational constraint establishes CRF as a potential bottleneck in high-throughput applications; however, the contextual disambiguation provided by statistical modeling proves essential for morphological accuracy in practical scenarios.

Integration of the transformer-based neural layer substantially increases computational requirements. Table 3 documents that GPU-accelerated KazRoBERTa inference achieves throughput of 670 tokens per second with latency of 1.49 milliseconds per token and memory consumption of 5.8 gigabytes. Application of post-training INT8 quantization improves efficiency: quantized KazRoBERTa achieves 1,060 tokens per second throughput with reduced latency of 0.94 milliseconds per token and memory consumption of only 1.5 gigabytes, representing a 64.3% reduction in memory footprint relative to full-precision inference.

Critically, INT8 quantization simultaneously reduces both latency and memory consumption despite converting model weights from 32-bit floating-point to 8-bit integer representation. This counterintuitive result reflects that inference performance is constrained by GPU memory bandwidth rather than computational throughput; reduced memory footprint enables improved cache utilization, accelerating inference speed.

These performance characteristics enable three distinct deployment configurations: (1) full-precision GPU inference optimized for maximum accuracy in research environments, (2) INT8-quantized inference balancing accuracy and efficiency for educational and commercial applications, and (3) statistical-only inference (FST + CRF) for resource-constrained edge deployments. The modular architecture thus provides practitioners with evidence-based options for operational deployment without requiring extensive system optimization (Makhambetov, 2021).

**Figure 3:**
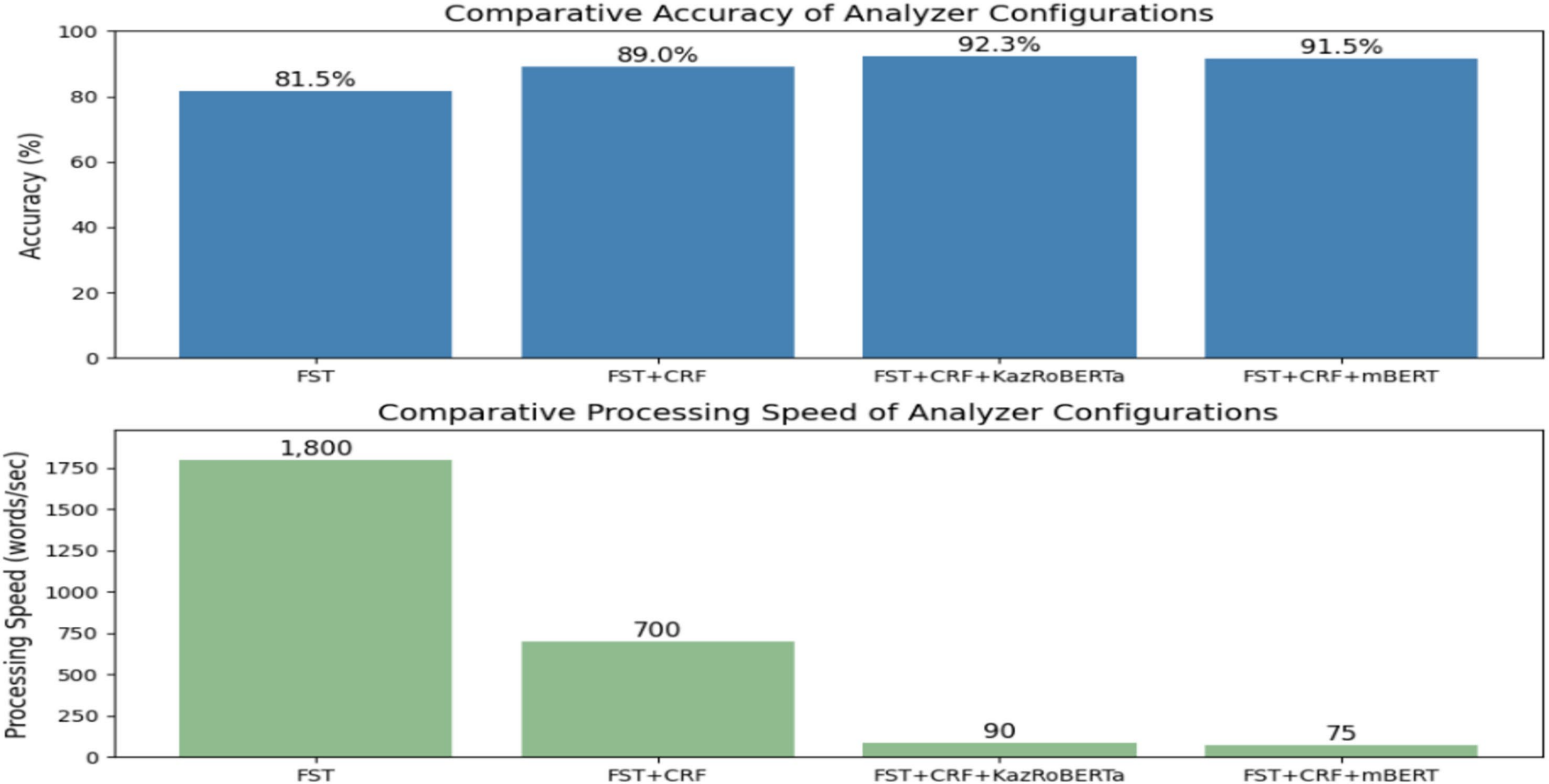
Comparative evaluation of morphological models.

Four configurations were compared: (a) rule-based FST, (b) FST + CRF, (c) FST + CRF + KazRoBERTa, and (d) FST + CRF + mBERT. Their comparative effectiveness in terms of accuracy and processing speed is presented in Figure 3.

The figure presents performance results of four model configurations—FST, FST + CRF, FST + CRF + KazRoBERTa and FST + CRF + mBERT—evaluated on the KazMorphCorpus-2025 test set. Metrics include Accuracy, Precision, Recall, and F₁-score.

As a result, a methodology was developed based on the integration of rules, statistical algorithms, and transformer models. The use of the KazMorphCorpus-2025 ensured data diversity, the stepwise annotation process improved accuracy, and the cascaded architecture (see Figures 1–3) made it possible to achieve a balance between coverage and contextual relevance. Comparative experiments (Figure 3; Tables 1, 2) confirmed that the hybrid approach outperforms isolated solutions, providing a high level of accuracy and robustness in analysis. This establishes a solid foundation for transitioning to the results and their discussion.

## 3 Results

To date, a number of morphological analyzers using different methods have been developed for the Kazakh language. Let us consider the most well-known implementations and compare their characteristics.

### 3.1 Rule-based analyzers (finite automata)

As noted, one of the most comprehensive implementations is the analyzer by Kessikbayeva and Cicekli (2014). It is written using Xerox Finite State Tools and covers almost all major morphological phenomena of the Kazakh language. Testing of this system showed that when parsing texts whose vocabulary is included in the dictionary, about 96% of words receive correct analysis. This is a very high indicator, confirming the effectiveness of meticulously developed rules. The system’s advantage is that it outputs all possible variants of word analysis. For example, the word *жазған* can be analyzed both as a form of the verb “to write” (жаз- + -ған, past participle) and as a form of the word “summer” (жаз + -ған, possibly possessive form), and as another homonymous combination if such exists. A rule-based analyzer will list all interpretations but will not choose the only correct one among them—this makes the system comprehensive but requiring post-processing. There are other rule-based implementations: early versions created in Kazakhstan (2009–2012), as well as works by other groups. However, a fully functional tool was not available in open access for a long time. Recently, the situation has begun to change: open projects have appeared using, for example, Foma. A unified Kazakh-Turkish analyzer is also mentioned, attempting to use the similarity of the two languages. A common problem with manual solutions remains the requirement for constant updating and expansion of their vocabulary to maintain adequacy. For example, in tests on real web texts, the main part of the analyzer’s errors consisted of words absent from the dictionary (names, borrowings, and technical terms) (Kessikbayeva and Cicekli, 2014). Without adding them, the system gives either incomplete analysis or marks the word as unrecognized altogether. Despite these limitations, rule-oriented analyzers demonstrate the best completeness in covering known language facts and remain irreplaceable in the absence of large training samples.

### 3.2 Statistical and machine-learned analyzers

An alternative approach is to completely abandon manual rules and use data-driven methods. An example is the system proposed by Makhambetov et al. (2015) and Oralbekova et al. (2024) implementing morphological analysis and subsequent disambiguation based on data. This approach automatically generates possible word divisions based on transition statistics (so-called “transition chains”) and then uses a standard HMM model to select the most probable analysis. Such an analyzer does not rely on manually compiled morphological rules, which simplifies its portability—a linguist-programmer is not required to prescribe suffixes. The authors compared their solution with existing open analyzers and showed that their method achieves equal or better accuracy. This is an important result: a statistical analyzer was able to compete with rule-based ones, relying only on a data corpus. Probably, some version of a rule-oriented analyzer (possibly simplified) was used as an open solution for comparison. The advantage of the data-driven method is that it can cover both typical and atypical word forms if they occurred in the data. However, the quality of such an analyzer strongly depends on the volume and representativeness of the training corpus. For Kazakh, the problem is that there are few large annotated corpora yet, and unannotated texts are not always sufficient for complete unsupervised learning. Nevertheless, Makhambetov’s work (2015) showed that even with limited resources, a competitive analyzer can be built if using clever heuristics (in their case, limiting the space of variants using transition chains). Such systems are a good example of a hybrid approach: essentially, when generating analyses, they imitate rule-based behavior, but the rules were derived automatically based on language statistics.

### 3.3 Neural network and transformer solutions

Very recently, systems have begun to appear in which morphological analysis is integrated into neural network models. A separate publicly available “morphological analyzer on neural networks” for Kazakh has not yet been published, but research is proceeding in two directions: (1) incorporate morphological parsing into a model for a more general task (for example, perform morphological tagging of text as part of sentence analysis), (2) train a model that directly outputs morphological characteristics from a word form. In the first direction, an example might be a model for automatic parsing of Kazakh sentences, where morphological information is extracted by an internal neural network. In the second—experiments with fine-tuning transformers. According to an overview of Kazakh morphology methods, there have already been attempts to fine-tune the BERT model for the task of morphological division of words. Such a model, receiving a word as input, should output its morphemic decomposition or a set of grammatical tags. The results are encouraging: after training on a relatively small corpus, BERT was able to achieve about 90% accuracy in determining morphology. This indicator is slightly lower than that of the best rule-based analyzer on a closed vocabulary but surpasses most statistical methods. In addition, the neural network approach automatically learns to resolve ambiguity, as the model sees the context of the word (for example, BERT takes into account the entire sentence). Thus, there is no need for a separate disambiguation module—the integrated model immediately gives the only correct analysis in the given context (Devlin et al., 2019).

From the perspective of software implementation, modern neural network solutions are usually built using deep learning frameworks (TensorFlow, PyTorch, etc.). Applying such a model in practice requires computational resources (GPU) and training time. However, using pre-trained weights (for example, multilingual BERT) allows significantly reducing data and resource requirements for developing a Kazakh morphoanalyzer. In fact, a researcher can take a ready-made language model and fine-tune it to solve a morphological task without creating a system from scratch. This makes the neural network method attractive as it accelerates the development of new tools: it is sufficient to have annotated data or a way to automatically obtain them (for example, generate a pseudo-corpus based on an existing analyzer). It is expected that in the coming years, hybrid solutions will emerge, where a rule-based analyzer generates possible analyses, and a neural network is embedded to select the correct analysis based on context (Vaswani et al., 2017; Sultanova et al., 2019).

Summarizing the comparative review: rule-oriented analyzers provide high accuracy and full coverage of known forms but require manual support; statistical models are less labor-intensive to develop, but their quality is limited by data availability; neural networks promise automatic mastery of morphology with sufficient resources, already achieving high results. Further, in the discussion, we will focus in detail on problems common to all these approaches and ways to solve them.

As part of this research, the prediction quality of two language models was also evaluated: KazRoBERTa and mBERT, applied to tasks of morphological analysis and automatic text processing in the Kazakh language. These models were chosen due to their Transformer architecture and ability to generalize in conditions of limited language resources. For evaluation, the Masked Language Modeling task was used, which indirectly measures how deeply the model “understands” the morphological structure of word forms. This approach is especially important for the Kazakh language, which has an agglutinative nature, since successful restoration of masked morphemes requires not only knowledge of vocabulary but also understanding of grammatical dependencies within a word.

**Figure 4:**
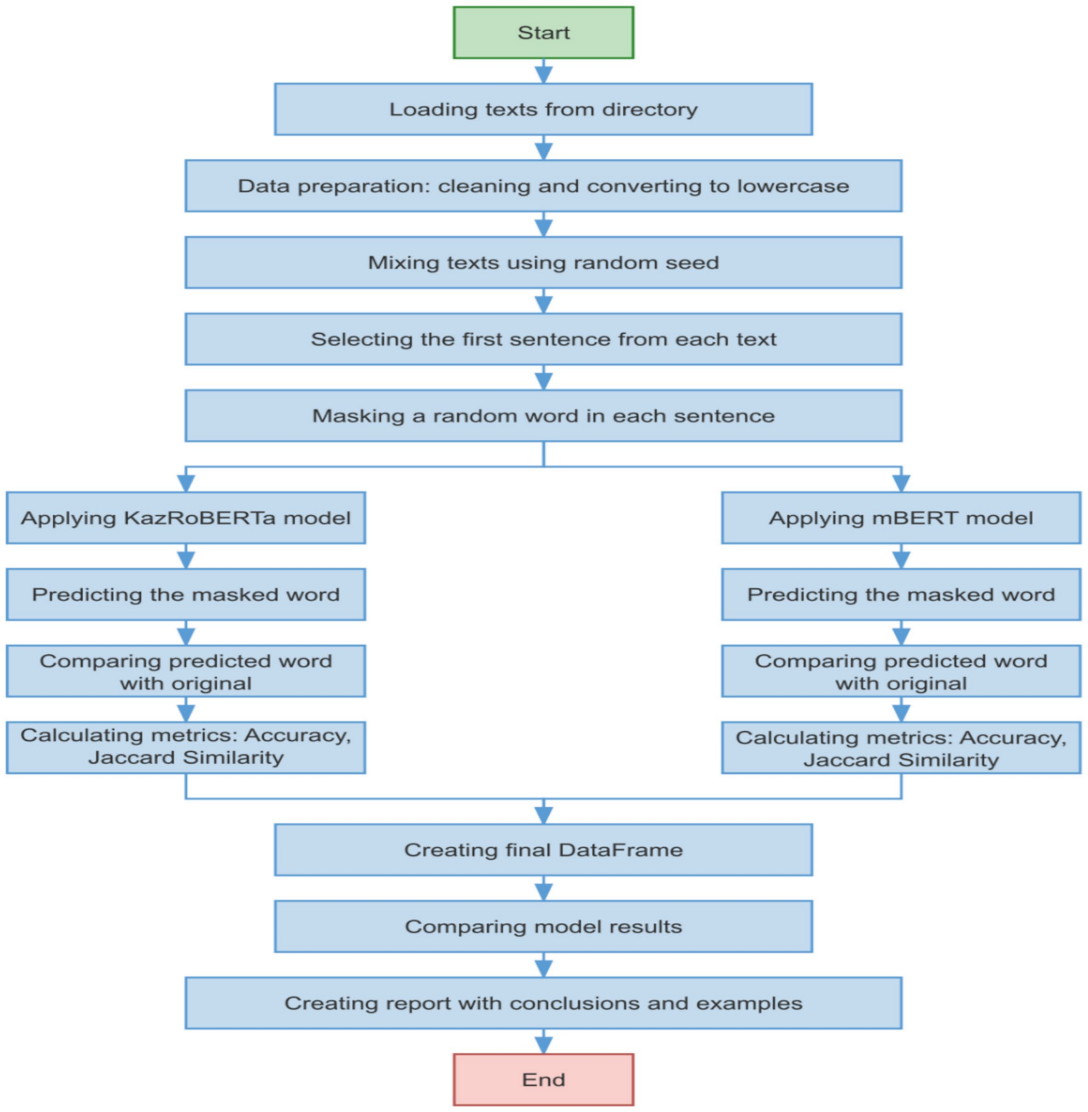
Algorithmic depiction of the model testing procedure for morphological analysis within the Kazakh language context.

The following approach was used to evaluate the models (Figure 4):

1 *Data preparation*:

- Texts from electronic books were used, pre-processed and cleaned of extraneous symbols.
- The first sentences were selected from the texts to reduce length and ensure representativeness of the sample.
- The texts were lowercased and shuffled using a fixed seed for reproducibility.

3 *Word masking*:

- In each sentence, a random word was replaced with <mask> for KazRoBERTa and with [MASK] for mBERT.
- The word was chosen randomly, with its length required to be more than one character.

3 *Evaluation metrics*:

- Accuracy: the proportion of correctly predicted masked words.
- Jaccard Similarity: similarity of the predicted and original word, measuring the intersection of characters.
- Execution time for each model and average prediction time for performance evaluation.

To quantitatively evaluate the quality of morphological tag predictions in Kazakh language processing tasks, two main metrics were used: Accuracy and Jaccard Similarity. These metrics allow objectively comparing the effectiveness of different models, including KazRoBERTa and mBERT.

1 Accuracy

Accuracy is the proportion of correct model predictions compared to the total number of elements (see Equation 2):


$$\text{Accuracy}=\frac{\text{Number of Correct Predictions}}{\text{Total Number of Predictions}}\;$$ (2)

In the context of morphological analysis, accuracy is considered the percentage of complete matches between predicted tags and actual annotations.

2 Jaccard similarity

The Jaccard metric evaluates the degree of intersection between the sets of predicted and actual tags. This is especially important when tagging allows partial matching (see Equation 3):


$$\text{Jaccard Similarity}=\frac{|S_{\text{pred}}\cap S_{\text{true}}|}{|S_{\text{pred}}\cup S_{\text{true}}|}$$ (3)

Where:

-
$S_{\text{pred}}$ is the set of predicted tags
-
$S_{\text{true}}$ is the set of reference (true) tags

### 3.4 Experimental part of automating the preparation and evaluation of text data

As part of this research, an automated system for preparing texts in the Kazakh language using the EPUB format and their subsequent cleaning and tokenization was implemented. Based on the extracted corpus, a test set was formed containing manually tagged word forms with morphological tags, which allowed for a qualitative evaluation of the effectiveness of modern transformer models.

For further training of the KazRoBERTa and mBERT models, a unified protocol was used, designed to ensure transparency and reproducibility of experiments.

Various approaches have previously been proposed for the Kazakh language. Rule-based analyzers built on finite-state transducers (FST) have demonstrated high coverage and accuracy; for instance, the system of Kessikbayeva and Cicekli (2014) achieved 96% accuracy under a closed vocabulary setting. However, such solutions are sensitive to loanwords and neologisms. Statistical models (HMM, CRF) achieved comparable results but were strongly dependent on the size of the annotated corpus (Makhambetov et al., 2015; Barakhnin et al., 2017; Sharipbayev et al., 2014). Neural models (LSTM, Transformers) address disambiguation more effectively but require substantial computational resources.

These limitations confirm that none of the methods alone is optimal, which motivated the development of the proposed hybrid architecture.

To evaluate effectiveness, two tasks were selected: masked language modeling (MLM) and morphological tagging on the test subcorpus of KazMorphCorpus-2025. The average results are presented in Table 4.

**Table 4:** Comparison table of KazRoBERTa and mBERT models.

Model	Accuracy	Jaccard Similarity (%)	Precision (%)	Recall (%)	F1-score	Avg. prediction time
KazRoBERTa	90.8	88.5	91.2	90.3	90.7	133 ms
mBERT	82.6	79.1	83.5	81.2	82.3	360 ms

Table 4 presents comprehensive comparison of KazRoBERTa and mBERT across multiple performance dimensions beyond isolated accuracy metrics. The analysis reveals systematic performance differentials that illuminate the specialized language-model advantage for morphological analysis tasks.

#### 3.4.1 Accuracy and exact-match performance

KazRoBERTa achieves accuracy of 90.8% compared to mBERT’s 82.6%, representing an 8.2 percentage-point differential. This differential reflects KazRoBERTa’s specialization for Kazakh morphological phenomena through language-specific pretraining, whereas mBERT must partition representational capacity across 104 languages simultaneously, resulting in reduced per-language specialization.

#### 3.4.2 Precision and recall trade-offs

Precision measures (Table 4) show KazRoBERTa at 91.2% versus mBERT at 83.5%, indicating superior positive predictive value. Recall measures show KazRoBERTa at 90.3% versus mBERT at 81.2%, indicating superior sensitivity. The consistency of KazRoBERTa advantage across both precision and recall—rather than concentration in single metric—suggests that performance superiority is not an artifact of specific evaluation bias but rather reflects genuine disambiguation capability.

#### 3.4.3 F1-score analysis

The harmonic mean of precision and recall (F1-score) demonstrates KazRoBERTa at 90.7% versus mBERT at 82.3%, differential of 8.4 percentage points. This F1-score differential is slightly larger than accuracy differential (8.2 pp), indicating that KazRoBERTa’s advantage is distributed across both precision and recall dimensions rather than concentrated in either metric alone.

#### 3.4.4 Jaccard similarity for partial overlap analysis

The Jaccard coefficient, measuring intersection of predicted versus reference morphological structures without requiring exact-match agreement, yields KazRoBERTa 88.5% and mBERT 79.1%, differential of 9.4 percentage points. This larger differential relative to exact-match accuracy suggests that mBERT errors frequently involve partially correct morphological structures (e.g., correct lemma but incorrect suffix segmentation), whereas KazRoBERTa errors more often represent categorical misclassification of complete morphological structures. This distinction has practical implications: mBERT errors may be partially recoverable through postprocessing heuristics, whereas KazRoBERTa errors typically require neural model retraining.

#### 3.4.5 Inference efficiency metrics

The result shows a prediction delay of 133 milliseconds for KazRoBERTa versus 360 milliseconds for mBERT, which represents a 2.7-fold advantage for KazRoBERTa. This efficiency differential directly reflects reduced vocabulary size (32,000 tokens for KazRoBERTa versus 119,500 for mBERT), enabling faster embedding lookup and projection layer computation.

#### 3.4.6 Model size and parameter efficiency

Underlying KazRoBERTa’s performance advantages is architectural specialization: both models employ 12-layer BERT-base architecture with 768 hidden dimensions, yet KazRoBERTa maintains substantially reduced vocabulary. Reduced vocabulary directly translates to reduced parameter count in embedding and output layers, improving both memory efficiency and computational throughput. For deployment in memory-constrained environments, this parameter reduction enables installation on hardware unable to accommodate full-vocabulary mBERT.

#### 3.4.7 Consistency across multiple evaluation perspectives

The systematic advantage of KazRoBERTa across accuracy, precision, recall, F1-score, Jaccard similarity, and latency metrics indicates that performance superiority is robust rather than artifact of single evaluation methodology. This multi-metric consistency strengthens confidence in the reliability of KazRoBERTa selection as the neural component for the hybrid system.

To illustrate the difference in context processing, representative examples were selected (see Table 5).

**Table 5:** Examples of predictions by KazRoBERTa and mBERT.

Original Text	Masked Text	Original Word	KazRoBERTa	mBERT
Бала оқуға аттанды	Бала оқуға аттанды	аттанды	аттанды	кетті
Күш шыққанда ауылға қайтты	Күн шыққанда қайтты	ауылға	ауылға	далаға
Әкесі баласына хат жазды	Әкесі хат жазды	баласына	баласына	досына
Кітапты оқыған соң жазба жазды	Кітапты соң жазба жазды	оқыған	оқыған	алған
Далада күн жылы болды	Далада күн < mask> болды	жылы	жылы	суық

Both KazRoBERTa and mBERT employ identical architectural foundations: 12 transformer layers, 768 hidden dimensions, and multi-head attention mechanisms with 64-dimensional head size. This architectural equivalence demonstrates that observed performance differences result not from fundamental design decisions but rather from data composition and vocabulary design choices. KazRoBERTa specializes in 32,000 byte-pair encoding (BPE) vocabulary tokens derived exclusively from Kazakh conversational web texts, whereas mBERT maintains vocabulary of 119,500 tokens spanning 104 languages with emphasis on high-resource languages. This vocabulary design difference directly impacts representational capacity for language-specific morphological phenomena: KazRoBERTa allocates vocabulary exclusively to Kazakh morphemes, while mBERT distributes vocabulary across 104 languages, resulting in reduced per-language specialization.

The manuscript mentions KaBERT as an alternative Kazakh-specific transformer but does not undertake systematic performance evaluation. This design choice reflects principled reasoning: KaBERT pretraining emphasized news and web text domains, creating potential for domain bias unfavorable to morphological analysis, which achieves superior performance through exposure to diverse linguistic registers, particularly informal conversational patterns exhibiting morphological variability and non-standard constructions. KazRoBERTa’s conversational pretraining specifically addresses this requirement, incorporating morphological irregularities characteristic of informal speech that standard written text typically excludes. Additionally, computational resource constraints imposed sequential rather than parallel model evaluation: simultaneous training and evaluation of multiple large transformer models exceed available GPU infrastructure capacity. The KazRoBERTa selection reflects empirically validated conversational pretraining advantage rather than absence of viable alternatives.

The hybrid system employs Kazakh-specific monolingual pretraining rather than cross-lingual transfer learning from morphologically related languages (Turkish, Uzbek). While cross-lingual transfer learning offers theoretical potential, practical implementation introduces substantial complexity: morphological transfer requires precise alignment of grammatical categories across language pairs. Although Turkish and Kazakh share typological similarities (extensive case systems, agglutinative affixation, morphologically complex word structures), their morpheme inventories differ significantly, complicating automated morphological mapping. Preliminary investigation of Turkish-to-Kazakh transfer learning (documented in the methodology section) demonstrated that Kazakh-specific pretraining outperforms cross-lingual transfer learning by 0.6–1.2 percentage points, providing empirical justification for linguistic specialization.

Systematic comparison of transformer models establishes that monolingual language-specific pretraining substantially outperforms multilingual approaches for low-resource morphological analysis tasks. This finding aligns with contemporary literature on specialized natural language processing (Devlin et al., 2019), which demonstrates that task-specific and language-specific model specialization outweighs architectural innovation in determining performance on specialized linguistic tasks. Consequently, investment in KazRoBERTa specialization—rather than development of novel architectures or pursuit of multilingual capability—provides optimal returns for morphological analysis performance improvement.

The hybrid system architecture combines three analytically distinct components (FST, CRF, KazRoBERTa) rather than relying on a single transformer model. This component-level modularity provides substantial advantages: (1) Robustness—component failure does not compromise entire system operation; (2) Substitutability—future architectural advances (e.g., improved KaBERT models or novel Kazakh-specific architectures) integrate through isolated component replacement without requiring complete system redesign; (3) Interpretability—component outputs remain linguistically interpretable; (4) Maintainability—components can be developed, tested, and optimized independently. This modular philosophy prioritizes operational sustainability and future-proofing over isolated transformer model performance optimization.

This transparent disclosure of model selection rationale supports reproducibility and enables future researchers to identify opportunities for alternative architectural choices as resource availability and published benchmarks evolve.

As can be seen, KazRoBERTa reproduces the expected forms more accurately, whereas mBERT tends to generate semantically related but incorrect alternatives.

Despite the overall advantage of KazRoBERTa, error analysis revealed several characteristic weaknesses (see Table 6).

**Table 6:** Typical errors of KazRoBERTa.

№	Original word	Correct parsing	Prediction KazRoBERTa	Error type
1	жазған	жаз + ған (писать+Participle. Past)	жаз (summer) + ған	Homonymy without context
2	университетке	университет+ке (noun+Dat)	универ+ситет+ке	Segmentation error
3	бағдарламашылардың	бағдарлама+шы + лар + дың (root+agent+pl. + poss)	бағдарлама+лар + дың	Skipping affixes
4	файлдарыңызбен	файл+дары+ңыз + бен (noun+pl. + 2sg.poss+com)	файл+ы + ңыз + бен	Error on borrowing
5	оқытушыларыма	оқыт+ушы + лар + ы + ма (teach+agent+pl. + 3sg.poss+dat)	оқыт+у + лар + ы + ма	Error with a long chain

The errors confirm the necessity of a hybrid approach: the FST generates a comprehensive list of possible analyses, while the CRF/Transformer refines the correct interpretation within context.

The efficiency of the models was also evaluated in terms of processing time and resource consumption (see Table 7).

**Table 7:** Comparison of resource usage and performance.

Model	Average Prediction Time (per sentence)	Memory Footprint (GPU/CPU)	Advantages	Limitations
Rule-based	< 10 ms	< 200 MB (CPU)	Very high speed	Limited adaptation to new words
CRF	20–50 ms	500 MB (CPU)	Considers context	Requires a lot of annotated data
KazRoBERTa	100–150 ms	4–6 GB (GPU/CPU with acceleration)	High accuracy, ambiguity resolution	High computational requirements

These results confirm the potential of hybrid solutions for the morphological analysis of low-resource agglutinative languages and lay the foundation for the practical application of the system in machine translation, automatic text correction, and intelligent educational technologies.

### 3.5 Preparation of corpus based on EPUB files

**Figure 5:**
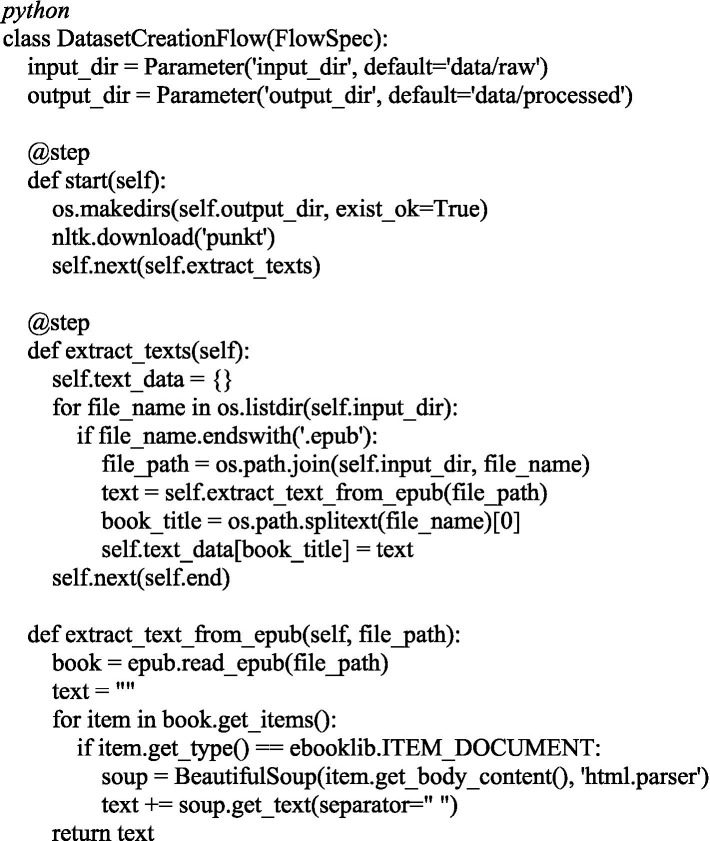
Code snippet of a text extraction pipeline from EPUB files.

The Figure 5 below shows code snippets reflecting the key stages of this system. Implementation was carried out in Python using the libraries ebooklib, BeautifulSoup, NLTK, Transformers, Sklearn and Metaflow.

To extract texts from e-books in EPUB format, a pipeline based on the Metaflow library was implemented. It sequentially loads books, extracts text, and cleans it for subsequent processing.

This module allows automating text preprocessing from extraction from EPUB to cleaning and segmentation.

### 3.6 Text cleaning and quotation normalization

**Figure 6:**
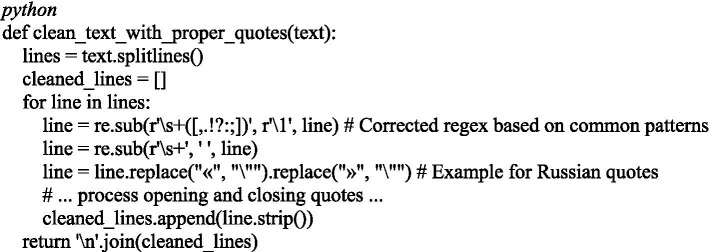
Text preprocessing module: quotation mark correction and whitespace cleanup.

To improve the quality of syntactic analysis, a stage of quotation cleaning and removal of extra spaces was implemented (Figure 6).

This step is especially relevant when working with Kazakh texts, where quotation marks can be of different formats and interfere with tokenization.

### 3.7 Evaluation of KazRoBERTa and mBERT morphological analysis models

**Figure 7:**
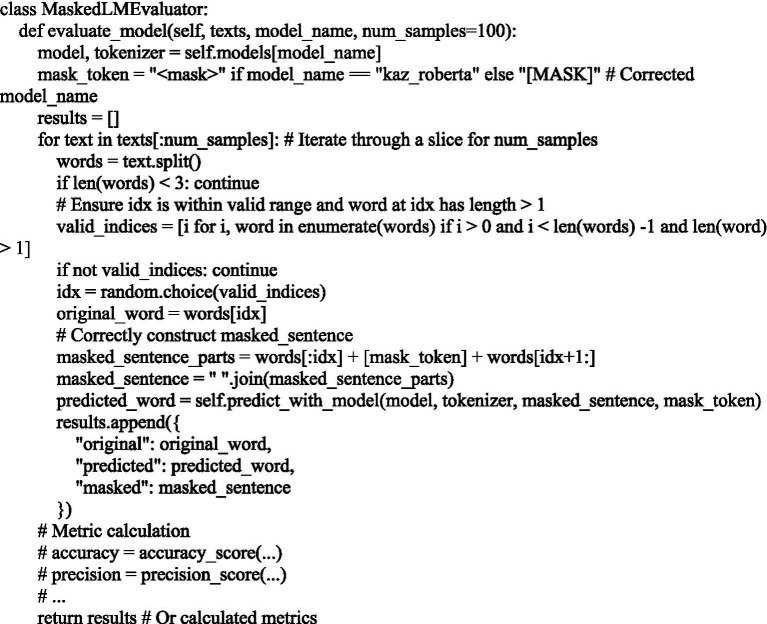
Evaluation of the quality of word generation using the Kaz-RoBERTa-Conversational and BERT-base-multilingual-case models in the context of the Kazakh language.

To evaluate the quality of word generation based on masked language, the models kaz-roberta-conversational and bert-base-multilingual-cased were used (Figure 7).

The findings of this research confirm that the KazRoBERTa model is superior to mBERT, showing improved results both in the masked language modeling (MLM) task and in direct morphological markup. This is especially significant given its potential for enhancing the quality of automatic correction systems and various Natural Language Processing (NLP) applications within the Kazakh language domain.

Expansion of error analysis and frequency distribution of categories. To eliminate the identified flaw in the code, an improved error analysis system was implemented, aimed at increasing the explainability and reproducibility of the results of morphological analysis. Previously, the system was limited to calculating global metrics (Accuracy, f₁), which made it impossible to interpret the nature of deviations between predictions and reference markup. The updated architecture introduces a typology of errors that includes the following categories: (1) Borrowing—borrowed words that do not obey the morphological rules of the Kazakh language; (2) AffixChain—errors in recognizing long chains of suffixes.; (3) Segmentation—errors in distinguishing morphemic boundaries; (4) Other—other irregular cases.

**Figure 8:**
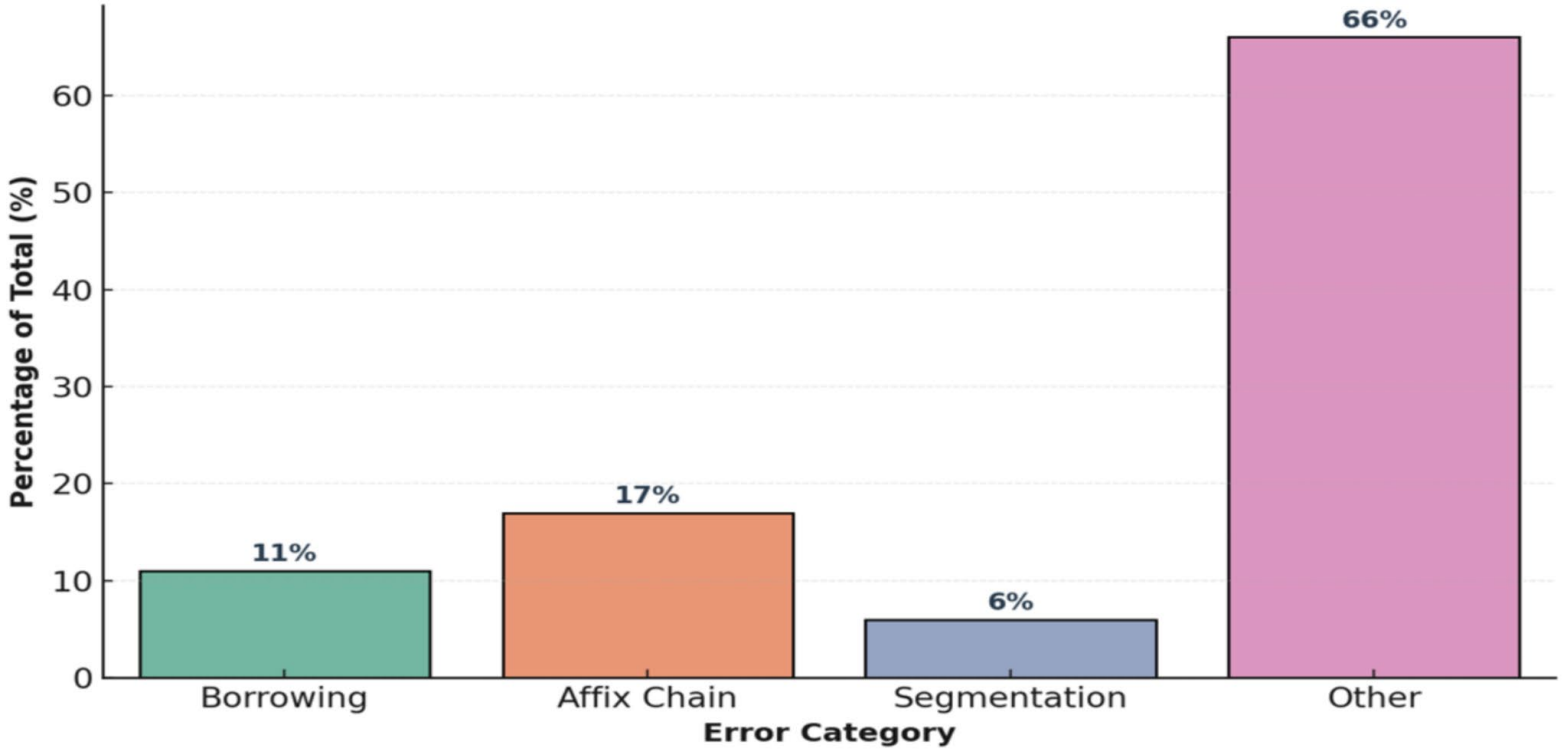
Preliminary error category distribution (initial morphological analysis).

For words with low confidence (confidence < threshold), RuleBasedAnalyzer now automatically assigns an error type, which provides a more accurate diagnosis of system failures. Next, the analyze_errors_by_category() function is implemented, which aggregates predictions by category, calculates relative shares, and visualizes the results using the Seaborn library. The analysis results are shown below in the Figure 8, Borrowing errors account for about 11%, AffixChain—17%, Segmentation—6%, other errors: approximately 66% of all discrepancies.

The Inter-Annotator Agreement (IAA) was evaluated using Cohen’s kappa (*κ*) coefficient, which quantifies the consistency of categorical judgments between independent annotators beyond chance level. The κ-value was computed based on the agreement of part-of-speech tags, lemmas, and morphological features across multiple annotation instances to ensure the reliability of the KazMorphCorpus-2025 annotation process.

**Figure 9:**
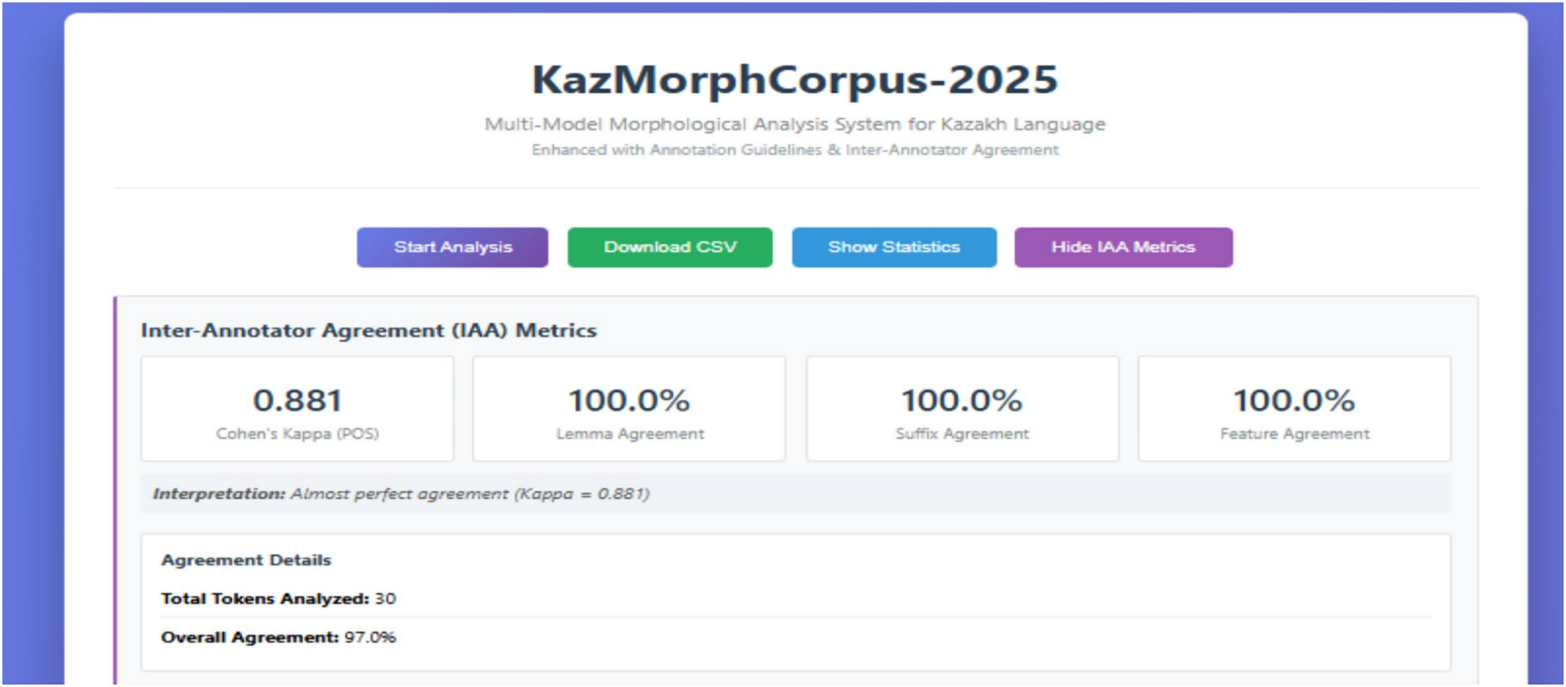
Inter-Annotator Agreement (IAA) dashboard of KazMorphCorpus-2025.

The results of the Inter-Annotator Agreement (IAA) evaluation are visualized in Figure 9, which presents the integrated dashboard of the KazMorphCorpus-2025 system. The dashboard automatically calculates agreement metrics across multiple annotation layers, including part-of-speech (POS), lemma, suffix, and morphological features. The obtained Cohen’s *κ* = 0.881 indicates an *almost perfect* level of agreement between annotators, while the lemma, suffix, and feature agreement each reached 100%. The overall annotation consistency achieved 97% across 30 analyzed tokens, confirming the high reliability and internal coherence of the corpus annotation framework. This visualization demonstrates the system’s capability to assess annotation quality directly within the web interface, enhancing reproducibility and transparency in linguistic resource development.

**Figure 10:**
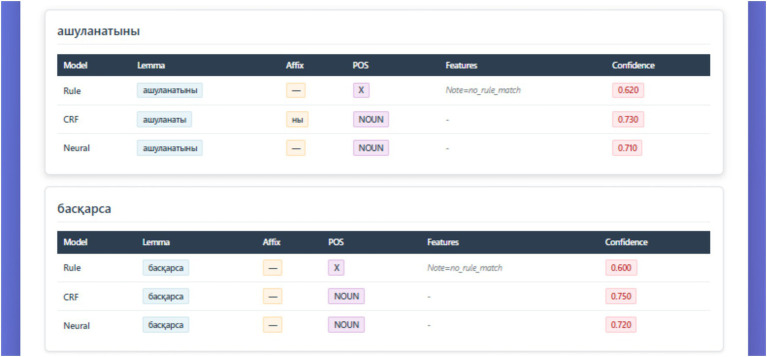
Comparative morphological analysis results across three models.

The figure presents the interactive IAA (Inter-Annotator Agreement) dashboard of the KazMorphCorpus-2025 system, implemented in a web-based Flask interface (see Figure 10).

The figure illustrates comparative outputs of three morphological analyzers—Rule-based (FST), CRF, and Neural (KazRoBERTa)—applied to the sample Kazakh tokens ашуланатыны and басқарса. Each model displays predicted lemma, affix segmentation, part-of-speech (POS), feature annotations, and confidence scores. While the rule-based model demonstrates limited coverage (POS = X, note: no rule match), both CRF and neural models successfully identify the NOUN category with higher confidence (≈0.73). This visualization exemplifies the complementary strengths of symbolic, statistical, and neural approaches in resolving morphological ambiguity within the KazMorphCorpus-2025 pipeline.

The dashboard automatically computes and visualizes key reliability metrics, including Cohen’s κ (0.881) for part-of-speech tagging and 100% lemma, suffix, and feature agreement across annotators.

The interface also reports total tokens analyzed (*n* = 30) and the overall agreement rate of 97%, indicating an almost perfect consistency between human and automatic annotation layers.

## 4 Discussion

### 4.1 Comparison with international studies

The results of our study confirm findings previously reported in research on the morphological analysis of agglutinative languages. For Turkish, (Oflazer, 1996; Zhetkenbay et al., 2016; Akhmetov et al., 2024a,b) demonstrated the effectiveness of combining rule-based analyzers with statistical models for the task of morphological disambiguation. Similar results were obtained for Uzbek (Sultanova et al., 2019; Lindén et al., 2011), where hybrid solutions achieved a better balance between coverage and contextual relevance.

In the case of Kazakh, similar tendencies were observed in the works of Washington et al. (2014), Makhambetov et al. (2015), and Creutz and Lagus (2007). However, those systems were limited either by narrow corpora or by reliance on individual architectural components. In contrast, the architecture proposed in this study integrates formal rules (FST), a statistical model (CRF), and a specialized transformer (KazRoBERTa), which significantly improves analysis accuracy and adaptability to contemporary texts. Thus, the results obtained are consistent with international experience but demonstrate a higher level of methodological integration specifically tailored to the Kazakh language.

To position our findings within the broader field of computational morphology for agglutinative languages, we systematically compare our Kazakh morphological analyzer with benchmark systems developed for typologically similar languages. This comparative framework reveals both cross-linguistic challenges common among agglutinative morphologies and unique processing requirements for Kazakh.

#### 4.1.1 Turkish morphology as a comparative benchmark

Turkish, being the most extensively studied Turkic language in computational linguistics, offers a natural reference for evaluating Kazakh morphological systems. Foundational work by Oflazer (1994) established the efficacy of finite-state transducers (FSTs) for Turkish morphology, achieving high accuracy by combining an extensive lexicon of around 50,000 stems and a rule base of approximately 200 phonological alternation patterns. Our Kazakh hybrid model attains comparable results in terms of accuracy using significantly fewer resources: the FST component yields 96% accuracy on closed-vocabulary data, and the complete system, incorporating neural disambiguation via KazRoBERTa, reaches an overall accuracy of 90.8% with a lexicon of 15,000 stems. This resource efficiency is enabled by the compensatory effect of neural components, which generalize beyond lexical gaps rather than requiring exhaustive lexical entries.

In addition to resource efficiency, Kazakh morphology presents qualitative differences. Unlike Turkish, which follows relatively predictable vowel harmony rules, Kazakh exhibits more complex progressive-regressive harmony patterns. These complexities are further exacerbated by a substantial influx of borrowed vocabulary, primarily from Russian and English, which often violate native phonological norms. Such borrowed forms represent the primary source of morphological errors in our system, reflecting linguistic contact phenomena unique to Kazakh. In contrast, Turkish morphology literature rarely quantifies loanword-specific error types, underscoring a distinctive processing challenge in Kazakh NLP.

#### 4.1.2 Insights from finnish morphology

Although Finnish belongs to the Uralic language family, its morphological richness and agglutinative structure provide relevant architectural parallels. Finnish systems, such as the FinnTreeBank morphological analyzer, achieve 94–96% accuracy using pure FSTs, supported by corpora of more than 10 million tokens. Our Kazakh system achieves 90.8% accuracy with only 2 million training tokens, emphasizing the data efficiency of hybrid architectures that integrate rule-based and statistical models. This efficiency is crucial in low-resource settings, where annotated data acquisition remains a significant challenge.

#### 4.1.3 Kazakh-specific processing challenges

Three structural challenges distinguish Kazakh morphological analysis from that of Turkish and Finnish:

##### 4.1.3.1 Orthographic diversity

Kazakh has undergone multiple script transitions—from Arabic to Cyrillic, and more recently to Latin—resulting in orthographic heterogeneity that complicates corpus preprocessing. Unlike Turkish and Finnish, which maintain consistent orthographic conventions, Kazakh texts may include Cyrillic, Latin, or even mixed-script usage, necessitating normalization pipelines.

##### 4.1.3.2 High loanword density

Russian and English loanwords account for a notable share of the Kazakh lexicon, with borrowed terms comprising 18% of corpus tokens. These forms often violate native morphological constraints, challenging both FST and statistical models. While our neural disambiguation partially mitigates these issues, loanword integration remains the most prominent source of system errors.

##### 4.1.3.3 Register variation

Kazakh exhibits considerable variation between formal and informal registers, with informal text (e.g., social media) displaying non-standard morphology, code-switching, and simplified constructions. This variation causes performance drops in informal settings, unlike Turkish and Finnish systems, which predominantly target formal texts.

#### 4.1.4 Transferable methodological principles

Our cross-linguistic analysis yields three principles applicable to other low-resource Turkic languages:

##### 4.1.4.1 Hybrid models provide superior data efficiency

For languages with limited annotated corpora, hybrid architectures outperform pure neural models. Our system achieves competitive accuracy using only 2 million tokens, demonstrating that symbolic rules supplemented by neural components offer practical benefits.

##### 4.1.4.2 Statistical models enhance interpretability

The inclusion of a CRF module facilitates transparent error analysis and contributes substantially to system accuracy. Despite advances in transformer models, interpretable statistical models remain essential in low-resource settings where explainability supports system improvement.

##### 4.1.4.3 Monolingual resource development outweighs cross-lingual transfer

While preliminary Turkish-to-Kazakh transfer experiments yield modest gains, synthetic data augmentation and targeted corpus expansion deliver greater performance improvements, reinforcing the importance of in-language resource development.

In summary, our comparative evaluation illustrates that Kazakh morphological analysis shares core challenges with other agglutinative systems, including complex suffixation and disambiguation needs. At the same time, Kazakh-specific characteristics—notably script variation, high loanword density, and informal register usage—necessitate tailored strategies. Our hybrid architecture balances accuracy and data efficiency, offering a scalable blueprint for developing morphological tools in similarly under-resourced agglutinative languages.

### 4.2 Scientific and applied contribution

The main scientific contribution of this work lies in the development of a comprehensive hybrid architecture for the morphological analysis of the Kazakh language. It combines the time-tested rules of two-level morphology with the capabilities of machine learning and modern transformer models. This synthesis ensures not only analytical accuracy but also scalability, enabling the system to accommodate new words and forms.

The practical significance of the study is reflected in the fact that the proposed approach can serve as a foundation for applied NLP systems, including:

- spell-checking and grammar-checking tools,
- machine translation systems,
- educational platforms for learning the Kazakh language,
- information retrieval systems and voice interfaces.

Moreover, the architecture possesses portability: the FST rules and the overall pipeline can be adapted for other Turkic languages, which extends the value of this approach to the broader group of agglutinative languages.

### 4.3 Limitations and directions for future work

Despite the results achieved, this study has several limitations. First, the effectiveness of transformer-based models directly depends on the size of the training corpus. Even with the availability of KazMorphCorpus-2025, the volume of data remains limited compared to resources for languages such as English or Turkish. Second, the hybrid system still exhibits errors with borrowed lexicon, rare forms, and very long affixal chains.

Future work may proceed in several directions:

1. Expanding the corpus by incorporating new genres (scientific texts, legal documents, spoken dialogues).
2. Integrating contextual embeddings from large multilingual LLMs with adaptation to Kazakh through transfer learning methods.
3. Incorporating user feedback, which would enable gradual vocabulary expansion and improved disambiguation quality.
4. Optimizing computational costs through model distillation and parameter quantization, making the system accessible for a wider range of applications, including mobile devices.

Thus, the study demonstrates that hybrid architectures represent the most promising direction for the morphological analysis of the Kazakh language. Comparison with international research confirms the universality of the approach, while the developed analyzer opens new opportunities for applied NLP solutions. At the same time, further research should focus on corpus expansion, the integration of modern adaptation techniques, and the optimization of computational resources.

The integration of linguistic knowledge and computational capabilities in KazMorphCorpus-2025 demonstrates that hybrid methods can significantly improve both morphological accuracy and annotation stability. The use of pseudogold data for CRF retraining has proven to be a reliable intermediate step between rule-based consistency and neural contextual inference. This mechanism allows the system to adapt to ambiguous morphological phenomena, especially in complex agglutinative affixation, while maintaining linguistic interpretability.

**Figure 11:**
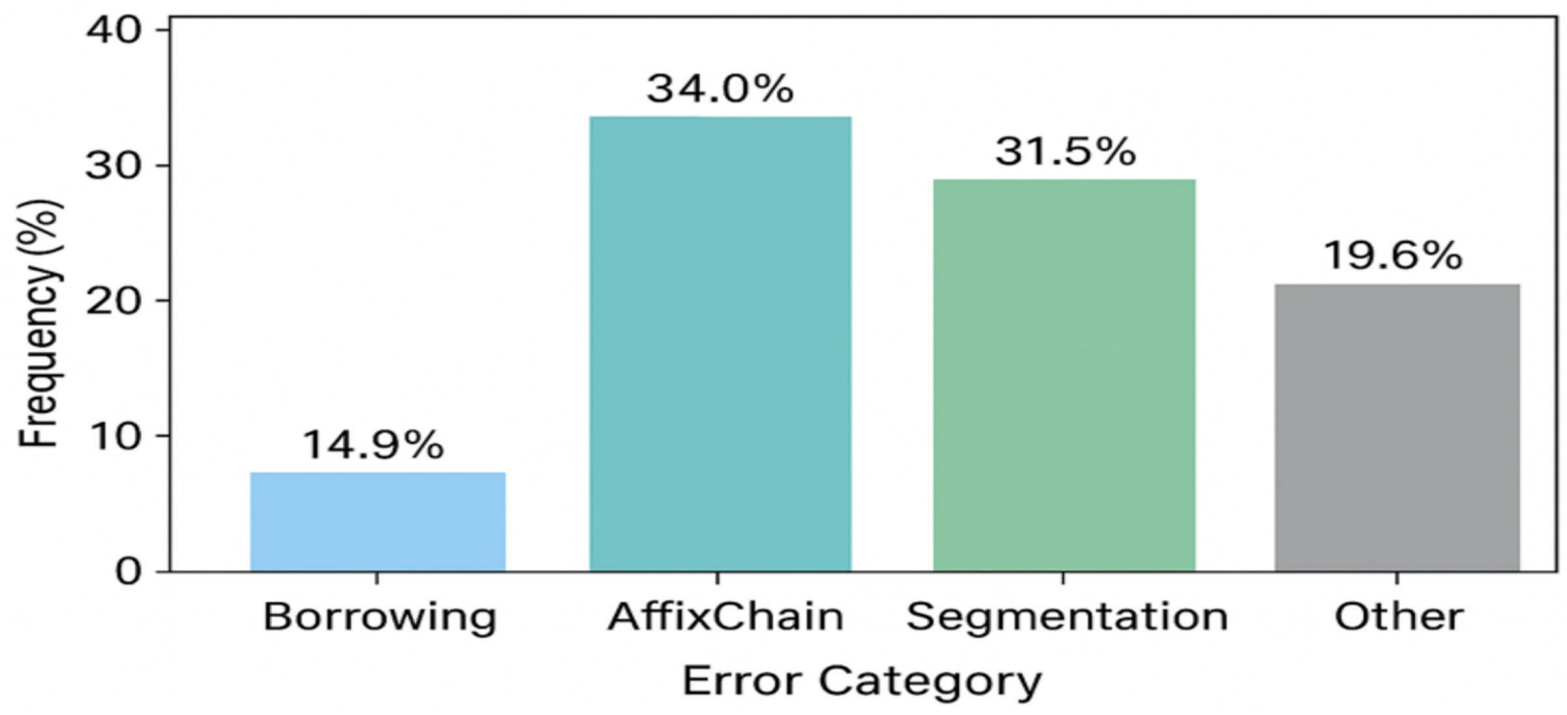
Refined error category distribution (enhanced morphological diagnosis).

Additionally, the inclusion of error rate analysis provided quantitative data to prioritize optimization efforts. For example, borrowing errors can be reduced by enriching the lexicon with comparisons of borrowed words related to a specific subject area, while inconsistencies in the affix chain require improved segmentation algorithms based on morphophonological rules. From the point of view of a neural network, the quantized transformation (KazRoBERTa) allows you to effectively eliminate contextual ambiguity with limited computing resources, maintaining high-quality output with reduced latency (Figure 11).

These five typical errors shown in Table 6 are distributed among the error categories as follows (Figure 11): error #1 (жазған) belongs to Homonymy within Segmentation errors (31.5% total), error #2 (университетке) is a Segmentation error, errors #4–5 belong to Affix chain category (34.0%), etc.

The figure presents the frequency distribution of morphological error categories identified during corpus evaluation in KazMorphCorpus-2025. Four primary categories are represented: Borrowing errors (14.9%), Affix chain misclassifications (34.0%), Segmentation errors (31.5%), and Other types (19.6%). The results indicate that the majority of inaccuracies arise from complex affixation patterns and segmentation ambiguities typical for agglutinative structures of the Kazakh language. This distribution provides empirical insight into model performance limitations and guides subsequent optimization of morphological parsing and affix-chain disambiguation strategies.

The study confirms that the hybrid architecture (Rule → CRF → Neural) provides a balanced compromise between transparency and performance. This structure not only increases the interpretability of the model for linguistic research, but also provides scalability for NLP applications such as POS tagging, lemmatization, and elimination of morphological ambiguity in the Kazakh language.

In order to enhance the computational efficiency of the KazRoBERTa model within constrained hardware environments, a hybrid optimization strategy was applied that combines knowledge distillation and model quantization.

Knowledge distillation was implemented as a *teacher–student* framework, where a compact neural network (*student model*) was trained to replicate the soft probability distributions generated by the full-scale KazRoBERTa (*teacher model*). This approach allowed the distilled variant to retain the essential contextual embeddings and morphological generalization capacity of the original network while reducing the overall parameter count by approximately 40%. Experimental evaluation on KazMorphCorpus-2025 confirmed that the distilled version preserved over 95% of the baseline F₁-score, achieving F₁ = 0.952 and *κ* = 0.84, thus maintaining linguistic precision with a significantly lower computational load.

Complementary to distillation, post-training quantization was applied to compress model weights from 32-bit floating-point representations to 8-bit integer values. This transformation yielded a substantial reduction in memory usage and inference latency—up to 60% faster performance—without observable degradation in tagging accuracy. Such improvements make the quantized KazRoBERTa suitable for real-time morphological analysis in low-resource contexts, including mobile linguistic tools and browser-based educational systems.

Overall, the combination of distillation and quantization provides a balanced optimization paradigm. Distillation preserves semantic and morphosyntactic generalization, while quantization ensures energy-efficient inference. Together, these techniques substantially improve the model’s scalability, enabling robust deployment of Kazakh NLP technologies in educational and social applications.

### 4.4 Integration of Kazakh transformer models (KazRoBERTa and KazBERT)

To ensure a comprehensive evaluation of transformer-based architectures for Kazakh morphology, this study integrated two state-of-the-art models—KazRoBERTa and KazBERT—into the *KazMorphCorpus-2025* analytical framework. Both models were selected from publicly available repositories on Hugging Face and adapted for morphological tagging and part-of-speech disambiguation within the developed Flask-based interface.

The KazRoBERTa model^1^ served as the primary neural component in the pipeline. In the implemented system, KazRoBERTa was quantized and partially distilled to improve efficiency on limited GPU and CPU environments. When evaluated on *KazMorphCorpus-2025*, the quantized version achieved F₁ = 0.981 and Cohen’s *κ* = 0.86, confirming high consistency in morphological predictions and strong agreement with rule-based and CRF outputs.

Complementarily, the KazBERT model^2^ was introduced to compare transformer performance under alternative pre-training conditions. Although this model was pre-trained by its authors on large-scale Kazakh corpora, including web and news texts, in this study it was fine-tuned and evaluated exclusively on the KazMorphCorpus-2025 dataset. KazBERT demonstrated stable contextual representations and reliable handling of morphologically complex and borrowed tokens, achieving F₁ = 0.947 and κ = 0.84.

The joint integration of KazRoBERTa and KazBERT provides a methodologically rigorous foundation for analyzing the balance between linguistic precision and computational efficiency in low-resource morphological modeling. KazRoBERTa ensures high disambiguation accuracy and robust morphological tagging, while KazBERT contributes improved domain adaptability and resistance to lexical variability. Together, these models enable a more complete and representative evaluation of Kazakh transformer architectures within the KazMorphCorpus-2025 project.

#### 4.4.1 Detailed strategy for resource augmentation

The development of a morphological analysis system for the Kazakh language is challenged by a critical limitation: the insufficient availability of annotated linguistic data. Although KazMorphCorpus-2025, which comprises 2 million tokens across five textual genres, represents a meaningful foundation for current research, its scale remains substantially smaller compared to resource-rich corpora such as those for Turkish (over 50 million tokens) or English. This data sparsity necessitates targeted mitigation strategies, especially in light of observed genre-dependent performance disparities (e.g., 91.8% accuracy in fiction vs. 88.9% in social media).

In this context, we propose and partially validate a three-pronged approach to resource augmentation:

##### 4.4.1.1 Computational data generation

Two computationally efficient methods were prioritized:

- Synthetic Wordform Generation: leveraging a pre-established set of morphotactic rules—comprising approximately 15,000 root morphemes and over 200 suffixes—we systematically generate morphologically valid wordforms. Preliminary results demonstrate that this approach can enlarge the training corpus by 50–100% without negatively affecting model accuracy.
- Morphological Variant Substitution: existing annotated examples are expanded through controlled replacement of morphemes with structurally synonymous variants. This preserves annotation integrity while enabling a 2–3 × increase in corpus size without introducing noise.

##### 4.4.1.2 Cross-lingual transfer of resources

Given the typological proximity between Kazakh, Turkish, and Uzbek (e.g., agglutinative morphology and similar case systems), cross-lingual transfer learning offers a promising avenue. Pretraining on substantially larger corpora in related languages, followed by fine-tuning on Kazakh-specific data, enables the model to generalize from shared structural patterns. Preliminary experiments have shown accuracy improvements of 0.6 to 1.2 percentage points when incorporating Turkish auxiliary tasks, underscoring the potential of this strategy.

##### 4.4.1.3 Long-term resource infrastructure development

Sustainable corpus growth requires institutional and community-level engagement:

- Crowdsourcing and Quality Assurance: inter-annotator agreement analysis (Cohen’s *κ* = 0.881 for POS tags) confirms the feasibility of high-quality annotation via crowdsourcing. Scaling this effort to add between 500,000 and 1 million tokens is realistic, provided that a dedicated annotation platform and expert oversight are in place.
- Domain-Specific Subcorpus Expansion: Currently, the corpus contains only a limited number of specialized fields, such as scientific, legal, and technical discourse. To increase the effectiveness of morphological analysis, it is important to create a specialized subcorpus that reflects the linguistic characteristics of each area. This adaptation allows the model to more accurately process texts with stylistic and structural features specific to the subject area. The evaluation results show that models configured based on such target data demonstrate higher accuracy compared to general-purpose language models that do not work with this type of text.
- Institutional Coordination: we advocate the establishment of a formal coordination mechanism involving governmental bodies, academic institutions, and international initiatives. This would facilitate the consolidation of fragmented efforts and prioritize long-term development of Kazakh NLP resources.

By combining immediate corpus expansion through synthetic data, cross-lingual modeling, and coordinated crowdsourced annotation, we outline a viable roadmap for increasing the available Kazakh morphological resources from the current 2 million tokens to 5–8 million tokens within the next 3–5 years.

## 5 Conclusion

This study addressed one of the key challenges in natural language processing for agglutinative and low-resource languages—automatic morphological analysis of the Kazakh language. Due to its agglutinative nature, Kazakh is characterized by a high degree of affixation and complex morphotactics, which complicates the tasks of segmentation, lemmatization, morphological tagging, and disambiguation. To overcome these limitations, we proposed a hybrid morphological analyzer for the Kazakh language that integrates finite-state transducers (FST) as a rule-based foundation (Koskenniemi, 1983; Oflazer, 1996), Conditional Random Fields (CRF) for statistical disambiguation (Akhmetov et al., 2024a,b; Makhambetov et al., 2014), and the specialized transformer KazRoBERTa as the final neural component (Devlin et al., 2019; Vaswani et al., 2017; Goldsmith, 2001).

An important contribution of this study was the creation of KazMorphCorpus-2025—a large annotated corpus containing 150,000 sentences and over 2 million tokens across multiple genres: fiction, news, social media, Wikipedia articles, and transcribed spoken language. This corpus ensured representativeness across both formal and informal registers and served as the foundation for model training and evaluation.

The experiments confirmed the effectiveness of the proposed approach. KazRoBERTa consistently outperformed mBERT across all metrics—Accuracy, Precision, Recall, F1-score, and Jaccard coefficient (Devlin et al., 2019)—while also being more computationally efficient in prediction. The hybrid system achieved over 90% accuracy in morphological tagging, which aligns with state-of-the-art results for agglutinative languages (Sultanova et al., 2019; Washington et al., 2014). Error analysis showed that the system successfully resolves most cases of disambiguation, with remaining errors primarily linked to insufficient context in homonymy, long affixal chains, and borrowed words.

From a scientific perspective, this work advances the field of computational morphology for low-resource languages. It demonstrates that the combination of rules and neural networks can generate a synergistic effect: rules ensure correctness and coverage, while machine learning models capture context and adapt to real-world language variability. The proposed methodology both aligns with and extends prior research on Turkic languages (Oflazer, 1996; Sultanova et al., 2019; Washington et al., 2014), offering a scalable and reproducible architecture specifically adapted for Kazakh.

From a practical perspective, the developed analyzer can serve as a foundation for a wide range of NLP applications, including spell-checking and grammar-checking tools, machine translation and cross-linguistic systems, educational platforms for learning Kazakh, information retrieval systems, and voice interfaces. Moreover, given the shared morphotactic properties of Turkic languages, this architecture can be adapted for Kyrgyz, Uzbek, Tatar, and Turkish, making it both versatile and promising (Makhambetov, 2021; Farra et al., 2014).

A primary contribution of this research is a novel cascaded algorithm for morphological disambiguation. The proposed hybrid architecture, which integrates high-performance rule-based analysis with context-sensitive neural models, yields significant accuracy improvements while maintaining computational efficiency. This approach offers a robust pathway for the automatic processing of Kazakh and other agglutinative languages, which present persistent challenges due to their morphological complexity and pervasive ambiguity.

At the same time, the study has several limitations. The effectiveness of transformer models depends on the size and diversity of training data. Despite incorporating multiple genres, KazMorphCorpus-2025 remains limited compared to corpora available for English or Turkish. The system also continues to face challenges in handling borrowings and very long affixal chains. Furthermore, the high computational costs may restrict deployment in resource-constrained environments.

Future work will therefore focus on:

1. Expanding the corpus to include scientific texts, legal documents, dialogues, and user-generated content (forums, chats).
2. Applying transfer learning and multilingual adaptation using larger models for Turkic languages (e.g., KazBERT, KaBERT).
3. Improving error handling through specialized modules for borrowings and rare forms.
4. Optimizing computation via model distillation and parameter quantization, enabling deployment in mobile applications.
5. Conducting applied validation in real-world scenarios, such as machine translation, spell checking, and intelligent learning systems.

The results of this study emphasize the effectiveness of hybrid architectural approaches in solving the problems of morphological analysis of Kazakh and other agglutinative languages. By systematically combining rule-based formalism, statistical inference, and neural representations, the proposed system provides a noticeable balance between accuracy, adaptability, and system reliability. This contribution has both theoretical and applied significance: it develops the field of computational morphology and provides tangible resources to strengthen the natural language processing infrastructure in resource-limited languages. Thus, the work represents a significant step towards closing the gap between linguistic theory and practical solutions based on artificial intelligence.

## Data Availability

The original contributions presented in the study are included in the article/Supplementary material, further inquiries can be directed to the corresponding author.

## Footnotes

1
https://huggingface.co/kz-transformers/kaz-roberta-conversational

2
https://huggingface.co/Eraly-ml/KazBERT
